# Definition of internal target volumes based on planar X‐ray fluoroscopic images for lung and hepatic stereotactic body radiation therapy. Comparison to inhale/exhale CT technique

**DOI:** 10.1002/acm2.12914

**Published:** 2020-05-30

**Authors:** David Sevillano, Luis Miguel Núñez, Margarita Chevalier, Feliciano García‐Vicente

**Affiliations:** ^1^ Department of Medical Physics Hospital Universitario Ramón y Cajal Madrid Spain; ^2^ Biomedical Engineering ETSIT Universidad Politécnica de Madrid Madrid Spain; ^3^ Department of Radiology, Rehabilitation and Physiotherapy Universidad Complutense de Madrid Madrid Spain

**Keywords:** fluoroscopic images, inhale/exhale CT, ITV definition, SBRT

## Abstract

**Purpose:**

To compare tumor motion amplitudes measured with 2D fluoroscopic images (FI) and with an inhale/exhale CT (IECT) technique

**Materials and methods:**

Tumor motion of 52 patients (39 lung patients and 13 liver patients) was obtained with both FI and IECT. For FI, tumor detection and tracking was performed by means of a software developed by the authors. Motion amplitude and, thus, internal target volume (ITV), were defined to cover the positions where the tumor spends 95% of the time. The algorithm was validated against two different respiratory motion phantoms. Motion amplitude in IECT was defined as the difference in the position of the centroid of the gross tumor volume in the image sets of both treatments.

**Results:**

Important differences exist when defining ITVs with FI and IECT. Overall, differences larger than 5 mm were obtained for 49%, 31%, and 9.6% of the patients in Superior‐Inferior (SI), Anterior‐Posterior (AP), and Lateral (LAT) directions, respectively. For tumor location, larger differences were found for tumors in the liver (73.6% SI, 27.3% AP, and 6.7% in LAT had differences larger than 5 mm), while tumors in the upper lobe benefitted less using FI (differences larger than 5 mm were only present in 27.6% (SI), 36.7% (AP), and 0% (LAT) of the patients).

**Conclusions:**

Use of FI with the linac built‐in CBCT system is feasible for ITV definition. Large differences between motion amplitudes detected with FI and IECT methods were found. The method presented in this work based on FI could represent an improvement in ITV definition compared to the method based on IECT due to FI permits tumor motion acquisition in a more realistic situation than IECT.

## 1 INTRODUCTION

Definition of Internal Target Volume (ITV) plays a main role in the accuracy of Stereotactic Body Radiation Therapy (SBRT) treatments. To define the ITV, a measurement of the displacements of the tumor during breathing is mandatory. Many methods allow the assessment of real tumor motion^1^: inhale/exhale CT (IECT) technique is feasible with a standard CT scanner, without extra devices^2^, ^3^; the slow scan method is an optional procedure included in some CT scanners^4^, ^5^, ^6^; planar fluoroscopy images can be produced with a digital flat panel forming part of an X‐ray system or with a CBCT^7^; and 4DCT and 4DCBCT are actually considered the state of the art in the detection of tumor movement by providing a complete set of images of the tumor throughout the breathing cycle.

Due to some drawbacks, the use of IECT may not be suited to measure tumor movement. On one side, the patient is not imaged in a normal breathing situation, which means that the inhalation and exhalation images are not representative of the actual movement of the tumor. On the other hand, it is dependent on the capacity of the patient to follow instructions correctly. Finally, it lacks information about the tumor's itinerary between the inhalation and exhalation phases. These limitations could be partially overcome with the slow‐scan procedure in which each slice is reconstructed over many respiratory cycles. This procedure allows to obtain an “average” image of the tumor. Nevertheless, it is not always easy to find the edges of the volume that encompass all tumor positions during a respiratory cycle due to the blurring associated with the tumor motion.

With 4DCT it is possible to define more realistic ITVs. In this case, the tumor is scanned along its trajectory throughout the breathing cycle by synchronizing the CT scanner to the patient's breathing with a special device capable of measuring the breathing phase. Then, the projections obtained can be combined in different datasets depending on the phase or the breathing amplitude.^8^, ^9^ 4DCT also allows to obtain the Maximum Intensity Projection (MIP)^10^, ^11^ that shows all tumor positions in only one image. 4DCT technique presents some shortcomings when compared to other methods like FI^12^ or 4DMRI.^13^, ^14^, ^15^ For example, 4DCT cannot detect inter‐ and intrafractional variations in the breathing pattern, which produces an insufficient representation of tumor movement. In addition, its high cost makes few departments have installed this type of equipment and its implementation is still limited even in developed countries.^16^ Thus, it would still be of interest to have some alternative methods to increase the accuracy in the measurement of tumor trajectories avoiding the acquisition of new and costly hardware.

In this work, we present a method based on planar fluoroscopic x‐ray images (FI) that permits realistic ITV using only a standard CBCT system. The results obtained are compared with those of IECT. Although many studies investigated the tumor movement with different devices and methods,^17^, ^18^, ^19^, ^20^, ^21^, ^22^, ^23^ as far as we know, there are no previous works comparing IECT with other methods for the definition of ITV. In addition, FI may be useful even when a 4DCT system is available since FI allows studying multiple breathing cycles and obtaining curves of position vs. time. Therefore, the shape of the breathing movement can be studied and decide the margins to be applied accordingly.

## 2 MATERIALS AND METHODS

### 2.1 Patients

A total of 52 patients were imaged with both FI and IECT. Treatment localizations were lung (39 patients, 12 in the upper lobe, 11 in the medial lobe, and 16 in the lower lobe) and liver (13 patients). All the patients were scanned and treated with their arms around the head, lying over a foam cradle and with a body mask performing abdominal compression.

Due to difficulties to locate tumors during daily treatment verification with CBCT, two to three 8‐shaped platinum pushable coils (Boston Scientific, Marlborough, MA, USA) were placed to liver patients inside or close to the tumor as fiducial markers. The markers were then used as tracking target in FI.

Treatments were planned with a Pinnacle 16.0 (Philips) treatment planning system (TPS). 3D conformal radiotherapy technique (3DCRT) was used for lung tumors except for the cases requiring a VMAT technique. This last technique was employed on all of the liver tumors. The Planning Target Volume encloses the ITV with a 5 mm margin. An Elekta (Elekta, Crawley, UK) C‐arm linac with an Agility MLC was used for treatment delivery.

### 2.2 Acquisition of planning CT scan

Each patient undergone three CT scans with a Philips Brilliance (Eindhoven, The Netherlands) system. One of them was a free breathing scan with 3 mm slice width for planning purposes; the other two were exhale and inhale scans where the patient was asked to stay in an exhale or inhale state during the image acquisition. These scans were focused only on the tumor volume with slice widths of 1.5 mm. Since the coordinate system is common for the three datasets, no registration was necessary. The tumor was contoured on each of the scans, and then transferred to the primary CT, where the ITV was defined as the sum of the contours of the tumor delineated on each of the three scans.

### 2.3 Acquisition of fluoroscopic images

FI were acquired with the XVI CBCT system (Elekta, Crawley, UK) of the treatment unit. The patients were placed in the unit with the system isocenter on the tumor position and identical setup to that of treatment. By placing the tumor at the isocenter is possible to measure displacements without scaling because the mm‐to‐pixel ratio (0.52 mm/pixel) is known.

Two projections, anteroposterior (AP), and lateral (LAT) were acquired. From the AP projection we could obtain information of Superior‐Inferior (SI) and LAT motion, while the LAT projection provides information of AP and SI motions.

We acquire a set of fluoroscopic images composed by 150 frames taken each 180 ms (total length of 27 s) that allowed the gathering of many breathing cycles.

### 2.4 Detection of tumor motion

The set of FI images were analyzed by means of an own software developed in MATLAB (MathWorks, Natick, MA, USA) to track the tumor motion along the images (tracking algorithm). The input of the tracking algorithm is a Region of Interest (ROI) with a rectangular shape that the user must select around the tumor in the frame (AP or LAT) where it is best visualized. This ROI is considered as the reference ROI. The ROI size is therefore dependent on the tumor size of each patient. The reference ROI is selected around the markers if fiducial marks are used and it is up to the user to select all fiducial markers or only a part of them. Next, the software performs a matching procedure to locate the tumor in the rest of the frames which is based on the calculation of the Normalized Cross‐Correlation (NCC) index. NCC is a widely used standard tool designed to detect features or similarities in intensity between two images of the same kind.^24^, ^25^, ^26^, ^27^ The NCC is calculated according to the expression^20^:

(1) $$NCC\left(u,v\right)=\frac{\sum_{x,y}\left[f\left(x,y\right)-\bar{f_{u,v}}\right]\left[t\left(x-u,y-v\right)-\bar{t}\right]}{{\left\{\sum_{x,y}{\left[f\left(x,y\right)-\bar{f_{u,v}}\right]}^{2}\sum_{x,y}{\left[t\left(x-u,y-v\right)-\bar{t}\right]}^{2}\right\}}^{0.5}}$$

where:

f(x, y) is the value of the pixel intensity at the (x, y) coordinates of a frame.

(u, v) represents the displacement of the reference ROI in the x and y directions, respectively.

$\bar{f_{u,v}}$ is the mean pixel value of the region under the reference ROI.

t(x, y) is the reference ROI and $\bar{t}$ is its mean value.

The numerator in (1) is a point‐by‐point convolution of the image and the reversed reference ROI that the algorithm calculates applying Fourier methods. The NCC output over each FI image is a matrix whose elements represent the NCC value at each position. The maximum value in the matrix corresponds to the detected tumor position in the frame. Manual correction was performed when the algorithm was not able to detect the tumor in any of the frames due to interferences with the anatomical structures in the image. The same process was repeated with all frame sets in order to obtain 3D data. The breathing motion curve for both directions (X and Y) is obtained from the positions of the NCC maximum in each frame.

### 2.5 Margin definition

In order to define an ITV with FI, we have considered that tumor motion curves were obtained in a reference system different from that of the planning CT. To match both reference systems, we have taken the origin in FI as the mean position of the tumor in the motion curves. The tumor position in the free breathing planning CT was taken as the mean position of the tumor. Once both reference systems are matched, the histogram representing the time the tumor spends at each position was obtained.

To avoid the effect of abnormal breathing cycles, the ITV was designed ensuring that it included the volume where the tumor spends 95% of the time. In this way, 2.5% of the extremal points were removed at each direction of the tumor position histograms, setting the margins there. Tumor position histograms of LAT or AP motion are obtained from AP or LAT projections, respectively, while data for SI motion are obtained from both projections. AP and LAT projections were centered around their mean positions and merged together to obtain a single dataset and a single position histogram associated with SI motion. Margins for SI directions were calculated from this histogram. Thus, we might expect margins in SI direction to be highly influenced by the projection in which detected SI motion is larger.

It is worth noting that this method implies applying asymmetric margins to the gross tumor volume (GTV) in the CT due to the breathing motion might not be symmetrical with respect to the mean position.

### 2.6 Test with breathing simulator phantoms

Accuracy of the employed algorithm was tested by measuring in FI the motion of two respiratory motion phantoms: Quasar phantom (Modus QA, USA) and a Synchrony® phantom (Accuray, Sunnyvale, CA, USA).

The Quasar phantom is designed as a motion table capable of performing different breathing curves in the superior/inferior direction sent from a controller software. On top of this table, a body shaped oval phantom with different geometric figures was placed. We imported four patient‐specific waveforms to the phantom that mimic breathing motion. Three of them had an amplitude of 10 mm and periods of 3, 4, and 5 s, while the other had an amplitude of 20 mm and a period of 4 s.

The Synchrony®^28^ phantom consists on a motion table equipped with fiducial marks that allow a periodic movement with an amplitude of 25 mm and varying periods selected by the user.

We used the algorithm above described for measuring in the FI frames the motion amplitudes associated with the phantom inserts or fiducial marks. The resulting amplitudes were compared with the nominal amplitude values to determine the uncertainties in the algorithm measurements.

### 2.7 Comparison between ITV margins obtained from fluoroscopic images and IECT

Motion amplitudes defined with FI were compared with those obtained with IECT for each patient. The differences were classified in three categories: equal or less to 3 mm, between 3 and 5 mm, and higher than 5 mm. The distributions of motion amplitudes for each treatment site and technique were characterized by its mean value and standard deviations. Two‐paired t test and F test were applied to check for significant differences.

## 3 RESULTS

### 3.1 Performance of the tracking software

The tracking software permitted to obtain ITV margins in an average time of 10 min per patient. The most important problem related to the algorithm performance was having to repeat the selection of the reference ROI to improve the tumor tracking. Manual corrections were needed when the tumor was in extremal positions within the breathing cycle or when the tumor is not found in the frame set. Usually, this correction had to be performed in one or two points per cycle. In the worst case, where the tracking fails for every cycle, and considering that we can measure up to seven or eight cycles per image set, the percentage of corrected points would be around 10%.

Anatomical structures appearing in LAT projections (mediastinum and spine bones) of FI can hamper the tumor detection in lung patients. In liver cases, LAT projections were more challenging and patient width was responsible for the algorithm fails in detecting fiducials. Due to this it was possible to obtain information of the LAT projection in 44 patients. For these cases, we obtained the difference between the margins obtained for the SI direction in AP and LAT views. The mean value and standard deviation of these differences were 0.3 mm (2.2 mm) for the whole dataset, 0.3 mm (2.3 mm) for lung patients, and 0.3 mm (2.2 mm) for liver patients.

### 3.2 Test with breathing simulator phantoms

**Fig. 1:**
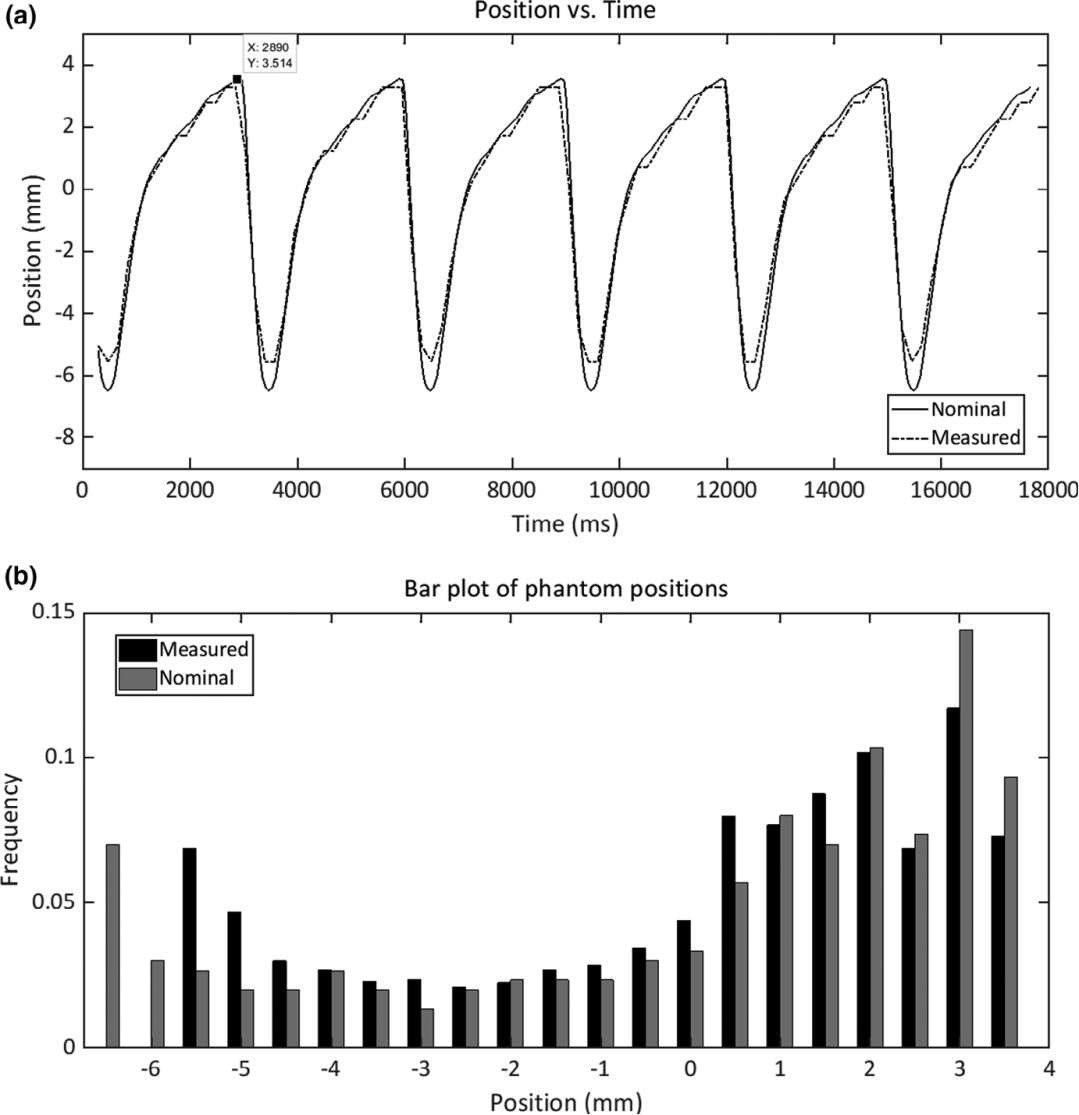
(a) Nominal (continuous line) and measured Quasar phantom positions (dashed line) vs. time in ms; (b) Probability distribution of the nominal positions of the Quasar phantom (grey columns) and those measured with the tracking algorithm (black columns).

Figure 1(a) shows the nominal motion curve imported to the Quasar phantom and that measured by our tracking algorithm for the case of nominal amplitude equal to 10 mm and period of 3 s. Measured motion amplitudes were 8.8 mm (absolute difference equal to 1.2 mm). For the case of nominal amplitude equal to 20 mm, the measured amplitude was 18.7 mm (absolute difference equal to 1.3 mm). These results did not show any dependency with the period.

Figure 1(b) shows the probability distribution of finding the tumor at each position according to the nominal and measured motion curves. Margins are obtained from these distributions by excluding the most extremal positions where the tumor spends 5% of the time. The effect of the amplitude underestimation detected in the measured motion curves had also its effect in calculated margins. For amplitude curves of 10 mm, margins of 8.7, 8.5, and 8.6 mm were recorded for the three different periods, while the true value was 9.7 mm. Margin for amplitude curve of 20 mm was 18.7 mm, compared with a theoretical value of 19.4 mm.

This fact may have a different impact in the calculation of margins from patients where amplitude and baseline shifts are not constant. With the aim of a more accurate assessment of the effect of the underestimation of detected amplitude in these cases, breathing curves from 10 patients were modified by decreasing tumor position around the inhale positions by a distance of 1 mm. Then, margins were calculated for modified and measured curves with the same method explained in section E. The mean difference in margins between real curves and modified curves was 0.5 mm, with a standard deviation of 0.3 mm. Differences obtained between curves depend on the uniformity of breathing amplitude and offset.

In the case of the Synchrony® phantom, the amplitude measured overestimates in 0.5 mm the nominal amplitude (25 mm) in contrast with the underestimation in the measured amplitudes found with the Quasar phantom.

The differences between the nominal and measured amplitudes for the Quasar phantom (Fig. 1a) come mainly from a loss of data in the inhale phase, where the tumor moves at a higher speed. We investigated the influence of the sampling frequency of fluoroscopic images in the underestimation of motion amplitude. The motion curve used by the software to control the tumor movement samples the tumor position each 10 ms. We randomly resampled the motion each 180 ms in a curve with an amplitude of 10 mm and a period of 3 s. We found that the maximum difference in amplitude was 0.03 mm.

### 3.3 Comparison between ITV margins obtained from fluoroscopic images and IECT

Table 1 shows the mean tumor motion amplitudes and their standard deviations at each direction for FI and IECT for the whole dataset and for each treatment location. The overall results revealed significant differences on the mean values of motion amplitude in the LAT and AP direction. From data desegregated by treatment locations, we can conclude that differences in the LAT directions come from the liver patients, while difference in AP direction corresponds mainly to those patients treated in the medium and lower lung lobes. The standard deviations of the amplitudes measured with FI are significantly lower than those from IECT when the overall distribution is considered. This result is also obtained for most of treatment sites, with the exception of the upper and lower lobes in LAT and SI directions.

**Table 1:** Mean values and standard deviations of tumor motion amplitude measured with inhale/exhale CT and with fluoroscopic images for each treatment site and at each direction.

Location	IECT						FI					
	LAT(mm)		AP(mm)		SI(mm)		LAT(mm)		AP(mm)		SI(mm)	
	M	SD	M	SD	M	SD	M	SD	M	SD	M	SD
Overall	3.5	2.7	6	4.8	10.7	8.1	2.1*	1.4*	3.3*	2.0*	8.7	5.7*
Overall lung	3.4	2.7	6.3	4.9	9.0	7.8	2.2*	1.5	3.1*	1.9*	8.5	6.1*
Lung — Upper Lobe	1.9	1.1	5.2	5.5	2.8	2.7	1.4	1.2	2.4	1.8*	3.5	3.0
Lung — Medium Lobe	5.1	3.9	8.4	6.0	10.3	8.1	2.5	1.2*	3.6*	1.7*	7.4	3.7*
Lung — Lower Lobe	3.2	2.0	5.6	3.8	12.6	8.2	2.5	1.9	3.4*	2.1*	12.6	6.7
Liver	3.7	2.5	5.3	4.1	15.1	6.9	1.9*	1.1*	4.1	2.1*	9.5*	3.6*

Values with * are those that show a significant difference (p<0.05) between fluoroscopic images and inhale/exhale CT.

By considering the overall dataset, we found that absolute differences between tumor motions detected with FI and IECT were larger than 5 mm for 49% of patients in the SI direction, for 31% of patients in the AP direction, and for only 9.6% of patients in the LAT direction. Thus, the treatment for half of the patients is highly dependent of the technique chosen to generate the ITV. Considering the treatment data by locations we found that, in the lung upper lobe, differences were larger than 5 mm for 27% (SI), 36.4% (AP), and 0% (LAT) of the patients; these percentages were in the lung medium lobe 40% (SI), 50% (AP), and 16.7% (LAT) and in the lung lower lobe, 41% (SI), 15.4% (AP), and 13.3% (LAT). Finally, for liver tumors, differences larger than 5 mm were for 73.3% (SI), 27.3% (AP), and 6.7% (LAT) of the patients.

**Fig. 2:**
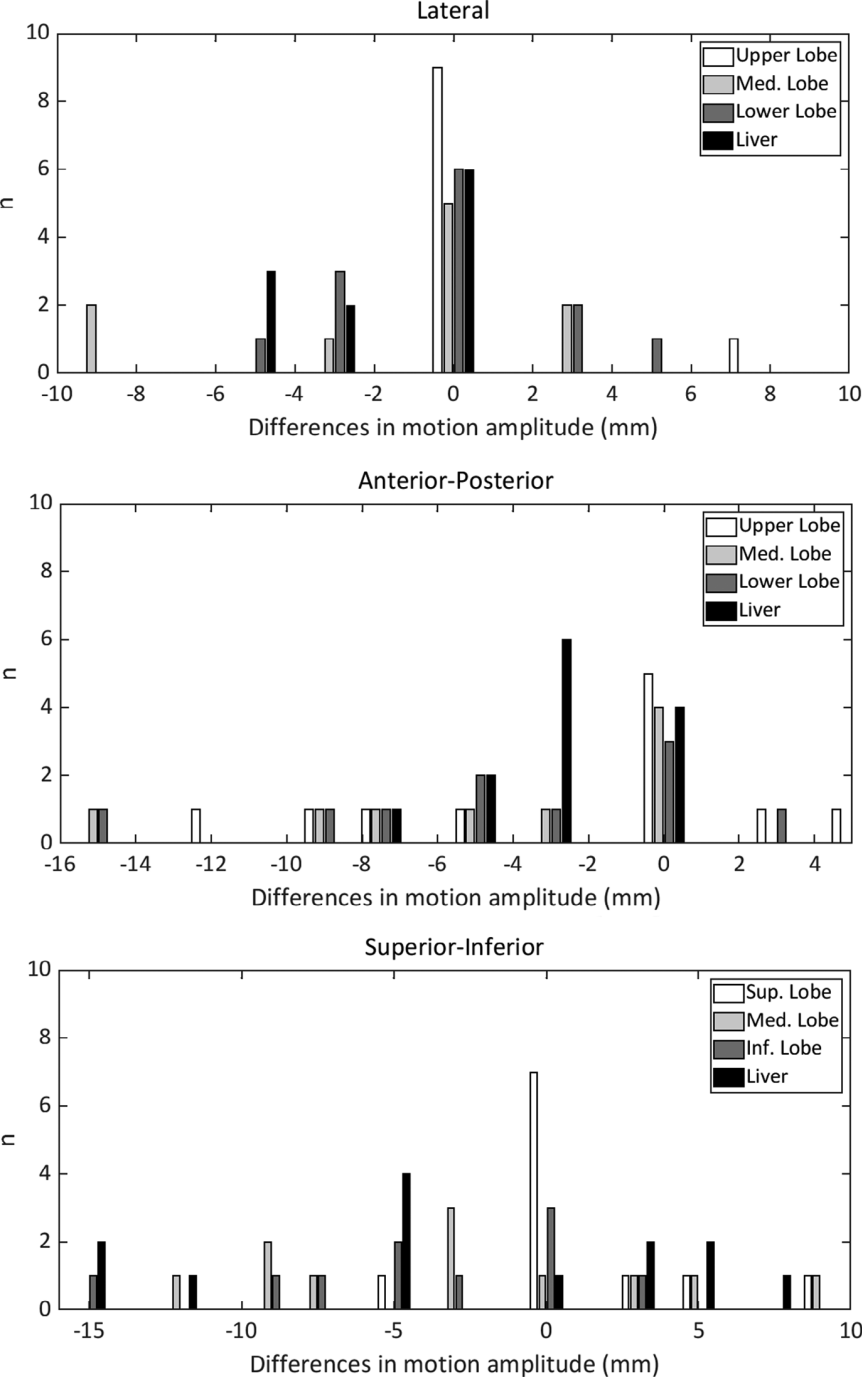
Bar plots of the differences in motion amplitudes (fluoroscopic images — inhale/exhale CT) measured at each direction by treatment location.

Bar plots of the differences between amplitudes obtained with IECT and FI for each treatment location are shown in Fig. 2. As can be seen, for lung tumors we found important differences in all directions except for the upper lobe. These differences were negatives (smaller amplitudes in FI than in IECT) in all directions except in SI direction. For lung, the proportion of patients having differences of more than +5 mm and less than ‐5 mm was similar (17.9% and 20.5%, respectively). For liver tumors, the percentage of differences larger than +5 mm (13%) in SI was smaller than those with negative sign (60%).

**Fig. 3:**
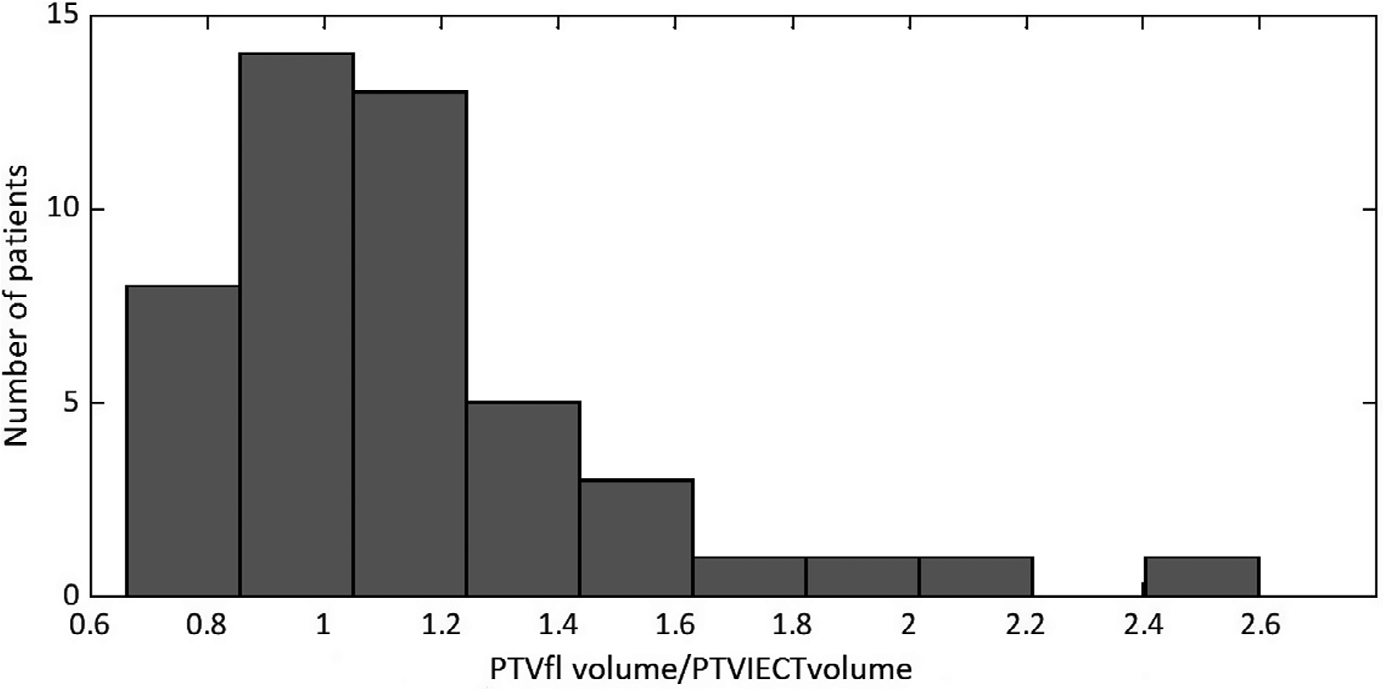
Bar plot of the values for the quotient of PTV volume obtained from fluoroscopic images (FI) and that obtained with inhale/exhale CT (IECT) for the sample of patients. The mean value of PTV volume with FI is 1.14 times higher than the one from IECT.

The quotient between PTV volumes obtained with FI and IECT is shown in Figure 3. As can be seen, for most patients, the PTV values obtained with FI images were higher than the IECT volumes despite the lower mean values of the movement amplitudes measured in FI. The mean value of the quotient of PTV volumes was 1.14 which implies an increasing around 15% in PTV volumes defined with FI. This increase can be due to two reasons: the first one is larger tumor motion amplitudes were measured with FI for some patients. In consequence, larger PTV volumes are obtained. The second one has to be with the Treatment Planning System (TPS) that only permits the creation of ellipsoidal ITVs whose axes are parallel to CT axis. Thus, these axes do not coincide to those of tumor motion when ITVs are created from FI images. This limitation of the TPS causes the increase in PTV volume.

## 4 DISCUSSION

The tracking software developed in this study was checked using two breathing simulator phantoms showing diverging outcomes for the algorithm performance. The algorithm measurements for the motion curves generated with the Synchrony phantom exceeded the nominal one by 0.5 mm. Measurements made with the Quasar phantom show the algorithm underestimates systematically the amplitude by about 1 mm. This result might be due to inaccuracies of the phantom itself, as sampling frequency was discarded to be the reason for this underestimation. However, the amplitude underestimation has a weak impact when breathing curves from patient are considered due to their high degree in variability.

The workflow presented in this work for ITV definition was successfully included in our clinical practice. As explained in the Results (subsection A), manual corrections were applied when tumor visibility was low or the tumor was in extremal positions of the breathing cycles. Although these corrections are not desirable, leading to a less efficient workflow, it was still possible to perform ITV definition in a period of time comparable to that used with the IECT method. The latter implies importing, registering, and contouring the tumor in the three CT sets. Some concerns could be raised on the effect of human intervention in the accuracy of the tracking software. However, it must be considered that the user has a relevant role in the whole process. Tumor detection and evaluation of software performance is the user's responsibility, as it is in many other areas in the radiotherapy process, so it is reasonable to rely on the user's criteria when a correction of tumor position is needed. Furthermore, the tests performed with the phantoms followed the same workflow as with patients, including manual corrections in some extremal position. The test results showed that the accuracy of the method is reasonably acceptable.

Comparisons between FI and IECT techniques yielded significant differences in mean values for the motion amplitudes in the AP direction for tumors in the lung medium and lower lobes, and in LAT and SI direction for liver tumors. Significant differences in the standard deviations associated with the motion amplitude measurements were also frequent, especially in the AP and SI directions. The differences in standard deviations correlate with the differences observed in the distributions of the measured amplitudes from both methods. These differences also justify the distributions shown in Fig. 2.

There were a large proportion of patients with differences between FI and IECT larger than 5 mm with a maximum percentage of a 73% for liver tumors in the SI direction. On the contrary, we found that these differences are smaller for patients treated in the upper lobe, even though we still find differences larger than 5 mm in the AP direction in 36% of patients.

We would have expected to find smaller amplitudes when measuring tumor motion with fluoroscopic images, as the breathing patterns are realistic and are not forced, unlike IECT. Nevertheless, we found that for an important proportion of patients, especially in the inferior and medium lobes, motion amplitudes detected with FI are larger.

From the obtained results, we can hypothesize two reasons to explain the substantial differences between IECT and in FI breathing patterns. Firstly, the significant differences in motion amplitudes in the AP direction for the medium and lower lung lobes suggest that the breathing pattern observed in IECT is forced. This fact yields to an overestimation of tumor motion and, more importantly, to a change in motion direction compared with normal breathing. In free breathing, movement in the AP direction is usually less significant than in SI direction due to diaphragmatic motion. On the other hand, during inhale/exhale patients tend to expand the thoracic cage instead of using the diaphragm, altering the movement of the tumor. Secondly, we could observe that, for many patients, motion amplitudes were much smaller in IECT. This can be ascribed to the fact that many patients do not follow correctly the instructions for inhale and exhale during CT acquisition. This effect occurs mainly in lung patients. For liver patients, the results show systematically smaller motion amplitudes in FI. This can be explained by the more attention paid by the staff when acquiring IECT for these patients, reducing the rate of patients not following instructions correctly.

In general, our FI results show greater motion amplitudes than those published by other authors, despite using abdominal compression to limit tumor movement.

For liver patients, we have mean motion amplitudes of 1.9, 4.1, and 9.5 mm in LAT, AP, and SI directions, while Shimohigashi et al.,^17^ using 4DCBCT and abdominal compression, obtained mean amplitudes of 1.7, 2.4, and 5.3 mm, respectively. In two studies^23^, ^24^ comparing tumor motion with abdominal compression and free breathing, they found that amplitudes for abdominal compression were similar to those of Simohigashi. et al.^17^ Hu et al.^23^ measured mean motion amplitudes of 2.9 mm in LAT, 2.3 mm in AP, and 5.3 mm in SI using 4DCT. Wunderink et al.,^24^ using FI, reported median amplitudes of 1.8 mm in LAT, 2.4 mm in AP, and 4.1 mm in SI. However, data gathered in free breathing in both studies are close to the values found in our work (3.1 mm in LAT, 2.9 mm in AP, and 9.9 mm in SI from Hu et al., and 1.2 mm in LAT, 4.1 mm in AP, and 9 mm in SI from Wunderink et al.).

For lung patients, Knybel et al.^20^ reported mean motion amplitudes of 2.2 mm, 2.8 mm, and 6.0mm in the LAT, AP, and SI directions, while our mean values in lung patients are, respectively, 2.1 mm, 3.4 mm, and 8.8 mm. In that study, using Cyberknife tumor tracking log files, no abdominal compression was applied. Despite this, mean amplitude values found by Knybel et al. were smaller than those obtained in this work. The mean values of tumor motion amplitudes reported by Sarudis et al.^21^ from 126 patients were similar to our results, but there is no mention to the presence of abdominal compression. For example, for the SI direction, a mean value of 3.1 mm was found for the upper lobe, 6.4 mm for the middle lobe, and 11.3 mm for the lower lobe, while we found values of 3.5, 7.4, and 12.6 mm, respectively. Same conclusions can be achieved if comparing with data from Bouilhol et al.^22^ Mampuya et al.^23^ reports longitudinal mean amplitudes of 20 mm for free breathing and 12.4 mm when applying abdominal compression. This last result is greater than that found in this work (mean value of 8.5 mm) in which only tumors with motion amplitudes greater than 8 mm were considered.

The comparisons with the studies from other authors suggest that an inefficient abdominal compression can explain the larger values obtained in our study.

The fact that differences found in SI motion from LAT and AP projections were the same for lung and liver suggests that our method succeeded to detect the same tumor structures in both projections for lung patients. Fiducial markers used in liver patients avoid confusion in tumor detection when they are visible. Also, the value of the standard deviation of the differences between projections obtained in this work (2.2 mm) is very similar to that of 1.5 mm reported by Suh et al.^29^ for Cyberknife patients. Thus, these differences could be explained by intrafraction variations in tumor motion and not by inaccuracies in the tracking algorithm.

## 5 CONCLUSIONS

The use of fluoroscopic images from the on‐board CBCT in the treatment allows for more realistic definitions of ITV compared to those obtained with IECT. Many problems with the use of IECT were detected in this work, such as tumor motion overestimation, due to excessively deep inspirations performed by the patient, or large underestimations due to incorrect fulfillment of inhale and exhale during CT acquisition.

A secondary finding of this work is that tumor motions obtained with abdominal compression are more correlated with data from other studies obtained without abdominal compression, suggesting a lack of efficacy of the employed abdominal compression method.

## CONFLICT OF INTERESTS

The author have no other relevant conflict of interests to disclose.
